# Rapid discrimination of strain-dependent fermentation characteristics among *Lactobacillus* strains by NMR-based metabolomics of fermented vegetable juice

**DOI:** 10.1371/journal.pone.0182229

**Published:** 2017-07-31

**Authors:** Satoru Tomita, Katsuichi Saito, Toshihide Nakamura, Yasuyo Sekiyama, Jun Kikuchi

**Affiliations:** 1 Food Research Institute, National Agriculture and Food Research Organization (NARO), Tsukuba, Japan; 2 RIKEN Center for Sustainable Resource Science, Yokohama, Japan; 3 Graduate School of Medical Life Science, Yokohama City University, Yokohama, Japan; 4 Graduate School of Bioagricultural Sciences and School of Agricultural Sciences, Nagoya University, Nagoya, Japan; Agricultural University of Athens, GREECE

## Abstract

In this study, we investigated the applicability of NMR-based metabolomics to discriminate strain-dependent fermentation characteristics of lactic acid bacteria (LAB), which are important microorganisms for fermented food production. To evaluate the discrimination capability, six type strains of *Lactobacillus* species and six additional *L*. *brevis* strains were used focusing on i) the difference between homo- and hetero-lactic fermentative species and ii) strain-dependent characteristics within *L*. *brevis*. Based on the differences in the metabolite profiles of fermented vegetable juices, non-targeted principal component analysis (PCA) clearly separated the samples into those inoculated with homo- and hetero-lactic fermentative species. The separation was primarily explained by the different levels of dominant metabolites (lactic acid, acetic acid, ethanol, and mannitol). Orthogonal partial least squares discrimination analysis, based on a regions-of-interest (ROIs) approach, revealed the contribution of low-abundance metabolites: acetoin, phenyllactic acid, *p*-hydroxyphenyllactic acid, glycerophosphocholine, and succinic acid for homolactic fermentation; and ornithine, tyramine, and γ-aminobutyric acid (GABA) for heterolactic fermentation. Furthermore, ROIs-based PCA of seven *L*. *brevis* strains separated their strain-dependent fermentation characteristics primarily based on their ability to utilize sucrose and citric acid, and convert glutamic acid and tyrosine into GABA and tyramine, respectively. In conclusion, NMR metabolomics successfully discriminated the fermentation characteristics of the tested strains and provided further information on metabolites responsible for these characteristics, which may impact the taste, aroma, and functional properties of fermented foods.

## Introduction

Fermented foods are produced globally using various ingredients, including vegetables, cereals, meats, fish, and dairy products, by utilizing the biological action of food-borne microorganisms [1]. Lactic acid bacteria (LAB) are microorganisms that are important in the food industry as they play a central role in producing numerous fermented foods and beverages such as fermented milks, cheeses, pickles, sourdough breads, and wines [2, 3]. The health-promoting effects of LAB, also known as probiotics, have led to the development of value-added foods.

Fermentation is a microbial metabolic process wherein chemical compounds are converted into other compounds known as metabolites. Activities of LAB during fermentation has been extensively studied [4–7]. Metabolism and resulting metabolites of LAB are responsible for the taste and aroma of fermented foods [1, 3, 8]. Furthermore, health-promoting compounds produced by LAB, such as γ-aminobutyric acid (GABA), provide additional value to fermented foods [9]. Recently, advanced analytical techniques have been utilized to identify metabolites responsible for the quality of fermented foods. Metabolomics focuses on the comprehensive measurement of metabolites in a biological system and has been used to analyze fermented foods involving LAB. For instance, previous metabolomics studies on fermented foods and beverages have highlighted the characteristics of red wines after malolactic fermentation by LAB [10], the impact of the proteolytic activity of LAB strains on set-yogurt [11, 12], and the role of metabolites as ripening-markers during the fermentation of Cheddar cheese [13].

Generally, a wide variety of LAB species or strains are involved in fermentation of traditional fermented foods. These food-borne bacteria exhibit diverse fermentation characteristics, and their metabolic activities can help modify and improve the sensory, nutritional, and functional qualities of fermented foods. To identify LAB strains possessing desirable metabolic activities, chemical analysis is required to detect target metabolites produced during fermentation. However, this process is often laborious and time-consuming, which limits the scale of experimental design (small number of target metabolites, test strains, and/or growth conditions). In recent studies, comprehensive metabolomic analysis discriminated the metabolite profiles of foods fermented with different LAB species or strains [14, 15], suggesting the potential of metabolomics for evaluating the fermentative characteristics of LAB strains. However, metabolomics has not been fully investigated for characterizing a wide variety of LAB strains.

In this study, we evaluated the potential of metabolomics as a method for rapid discrimination of fermentation characteristics of LAB strains by analyzing fermented vegetable juices. The environment inside fermented foods differs markedly from laboratory growth media owing to multiple factors such as limited and unbalanced nutrients, presence of various carbohydrates (carbon sources) and undigested components (proteins and dietary fibers), low initial pH, and presence of inhibitory compounds. Fermentation of vegetable juice reflects these conditions and can be a useful model of plant-based fermented food. Moreover, from a biochemical perspective, many LAB genera and species require rich and complex media because of their multiple auxotrophies for amino acids, vitamins, and nucleic acids [16]. Even strain-dependent attributes are often observed, for instance, in the carbohydrate assimilation ability of LAB [17]. Therefore, we expect the metabolite profile of fermented vegetable juices to exhibit strain-dependent patterns, enabling us to discriminate fermentation characteristics at the strain level.

In this study, we utilized nuclear magnetic resonance (NMR)-based metabolomics to comprehensively analyze fermented vegetable juices. NMR analysis has several advantages, including simplicity of sample preparation, rapidity of spectrum measurement, wide dynamic range, and reproducibility [18]. The analyses performed in this study successfully separated the tested strains based on species- and strain-dependent fermentation characteristics, revealing correlations with various compounds responsible for the taste, aroma, and functional properties of fermented foods. We describe the applicability of NMR-based metabolomics as a rapid discrimination method for *Lactobacillus* strains, and the possible impact of highlighted strain and its metabolites on the quality of fermented foods.

## Materials and methods

### Bacterial strains and culture conditions

Six type strains of *Lactobacillus* species and six additional *L*. *brevis* strains were selected to investigate the discrimination capability focusing on i) the difference between homo- and hetero-lactic fermentative species and ii) strain-dependent characteristics within *L*, *brevis*. The following 12 strains were obtained from NITE Biological Resource Center (www.nite.go.jp/en/nbrc/) in Japan: heterofermentative LAB strains, *L*. *brevis* NBRC 3345 (Lm1; = ATCC 8287), NBRC 3960 (Lm2), NBRC 12005 (Lm3), NBRC 12520 (Lm4), NBRC 13109 (Lm5), NBRC 13110 (Lm6), NBRC 107147^T^ (Lm7), *L*. *fermentum* NBRC 15885^T^ (Lm12); homofermentative LAB strains, *L*. *pentosus* NBRC 106467^T^ (Lm14), *L*. *plantarum* subsp. *argentratensis* NBRC 106468^T^ (Lm15), *L*. *plantarum* subsp. *plantarum* NBRC 15891^T^ (Lm23), and *L*. *rhamnosus* NBRC 3425^T^ (Lm29). The strains were grown in Difco Lactobacilli MRS Broth (Becton, Dickinson and Company, Franklin Lakes, NJ) at 30°C for 24 h and stored in 1.2% agar stubs of the same medium supplemented with 0.5% calcium carbonate.

### Preparation of fermented vegetable juices

Fermented vegetable juices were prepared using five commercially available vegetable juices (A–E) produced by two major beverage manufacturers in Japan (Kagome Co., Ltd., Nagoya, Japan; Kirin Beverage Co., Ltd., Tokyo, Japan). Juices C–E were 100% vegetable juices made from several dozen leaf and root vegetables, while juices A and B contained vegetable and fruit juice. None of the juices contained sweeteners, salts, or preservatives. Nutritional composition of the vegetable juice products is indicated in S1 Table. The juices were diluted to 50% concentration with sterilized water and inoculated with precultures (5% v/v) of the strains tested. The inoculated juices were fermented by incubating at 30°C over three durations (3 days, 1 week, or 3 weeks) considering different periods required for production of various fermented foods. Samples from these three incubation periods were prepared in independent inoculation batches. Additionally, juices inoculated with sterilized water as controls. In total, we prepared 195 samples. After incubation, the juices were centrifuged at 17,400 × *g* for 5 min at room temperature (25°C). The supernatants were collected and stored at -20°C until NMR spectral analysis.

### NMR spectroscopy

NMR analytical samples were prepared as described previously [19]. To analyze water-soluble metabolites in the fermented juices, we used a deuterium oxide (D_2_O)-based potassium phosphate buffer (KPi) consisting of 125 mM K_2_HPO_4_/KH_2_PO_4_ (pH 7.0) and 1.25 mM 2,2-dimethyl-2-silapentane-5-sulfonate sodium salt (DSS; Sigma-Aldrich, St. Louis, MO) in D_2_O (99.9% D; Cambridge Isotope Laboratories, Andover MA). Briefly, 140 μL supernatant of each sample was diluted with 560 μL of KPi and, after centrifugation, the clear supernatant was transferred to an NMR sample tube (5.0 mm O.D. × 103.5 mm; Norell, Landisville, NJ). NMR spectra were recorded on an Avance-500 spectrometer (Bruker BioSpin, Karlsruhe, Germany) equipped with a carbon/proton CPDUL CryoProbe (Bruker BioSpin) and a SampleJet automatic sample changer (Bruker BioSpin) using the automated software IconNMR (Bruker BioSpin). ^1^H NMR spectra for metabolomic analysis were acquired at 298 K using the Bruker pulse program zgpr, as described previously [19]. Given these acquisition conditions, the measurement time of each sample was approximately 15 min, resulting in 2 days of measurement for all 195 samples.

For metabolite annotation, two-dimensional (2D) NMR spectra, including double quantum-filtered correlated spectroscopy (DQF-COSY), totally correlated spectroscopy (TOCSY), ^1^H–^13^C heteronuclear single quantum coherence (HSQC), and ^1^H–^13^C heteronuclear multiple-bond connectivity (HMBC), were recorded on the same instrument. Where appropriate, 2D NMR spectra were measured on an Avance-800 spectrometer (Bruker BioSpin) at proton and carbon frequencies of 800.33 and 201.24 MHz, respectively. The SpinAssign program at the PRIMe web service (Platform for RIKEN Metabolomics: http://prime.psc.riken.jp/) was used for annotating metabolite signals in HSQC spectra. Peak tables of HSQC spectra were generated using NMRPipe and NMRDraw as described previously [19]. Metabolite annotation was carried out by spectral comparison with standard compounds. Two online spectrum databases, the Human Metabolomics Database (http://www.hmdb.ca/) and the Biological Magnetic Resonance Data Bank (http://www.bmrb.wisc.edu/), were also used.

### NMR-based metabolic profiling

Prior to dataset preparation, we examined the ^1^H NMR spectra for chemical shift fluctuations among samples to confirm the absence of misalignments that could affect subsequent multivariate analyses. Two spectral subdivision methods were used for dataset preparation. For exploratory, non-targeted analysis, the dataset was generated as described previously [19]. Briefly, processed ^1^H NMR spectra were subdivided into 0.04-ppm width integral regions (buckets) in the 10.0–0.50 ppm spectral range. Nine buckets containing residual water signal (5.04–4.68 ppm) were excluded from the analysis, providing a dataset comprising 229 buckets. The integral value of methyl signals at 0 ppm of DSS was used for normalization. The second subdivision method was based on a regions-of-interest (ROIs) approach [20]. Here, we subdivided the spectra into manually specified spectral ranges containing NMR signal of single compound. Signals derived from unannotated metabolites were also specified. Based on 180 spectra, excluding controls, this manual subdivision provided 101 integrals of annotated and unannotated metabolite signals. Subsequently, to reduce the large dynamic range among integrals and to focus on low-abundance metabolites, integral values were normalized by standard score (*Z*-score) transformation across the 180 samples.

Principal component analysis (PCA) and orthogonal partial least squares discrimination analysis (OPLS-DA) were carried out using the SIMCA software (ver. 14.0.0.1359; Umetrics, Umeå, Sweden). Pareto scaling and mean centering were applied to the dataset for non-targeted and ROIs-based analyses, respectively. Models generated by OPLS-DA were evaluated by leave-one-out cross-validation and permutation test (n = 999). The software automatically selected the optimum number of latent variables.

## Results and discussion

### Preparation of fermented juice by LAB

The pH of all samples decreased after fermentation. The initial pH was approximately 4.30 for juices C and D and 4.00 for juices A, B, and E. Control samples without LAB inoculation showed almost no change in pH (< ±0.1) during incubation for 3 weeks. After inoculation, the pH of fermented juices decreased substantially over 3 days, and maintained a low level up to 3 weeks. For instance, the pH of juice C during fermentation with strain Lm1 decreased from pH 4.24 (initial) to 3.61 (3 days), 3.59 (1 week), and 3.52 (3 weeks). The terminal pH of samples with homofermentative strains (pH 3.16–3.30) was relatively lower than that of samples with heterofermentative strains (3.51–3.70). This difference in pH suggested that the metabolite profile of fermented vegetable juices differed between the two fermentative types.

### Metabolite annotation by NMR analysis

The following 53 metabolites were annotated by NMR analysis in fermented and control vegetable juices: carbohydrates (sucrose [Suc], glucose [Glc], fructose [Fru], galactose, trehalose, mannose, glycerol, mannitol, *myo*-inositol, *scyllo*-inositol, and galacturonic acid); organic acids (lactic acid [LacA], malic acid [MalA], citric acid [CitA], succinic acid [SucA], acetic acid [HOAc], fumaric acid [FumA], formic acid [ForA], tartaric acid, quinic acid, phenyllactic acid [PLA], 4-hydroxyphenyllactic acid [HPLA], and 2-hydroxyisovaleric acid [HIVA]); amino acids (alanine, arginine [Arg], asparagine [Asn], aspartic acid [Asp], glutamine, glutamic acid [Glu], phenylalanine [Phe], proline, serine, threonine, glycine, histidine, valine [Val], leucine, isoleucine, and tyrosine [Tyr]); others (GABA, ornithine [Orn], pyroglutamic acid, tyramine, ethanol [EtOH], methanol, *tert*-butanol [*t*BuOH], uracil, uridine monophosphate [UMP], choline, glycerophosphocholine [GPC], acetoin, dihydroxyacetone [DHA], and *NNN*-trimethylglycine). Representative ^1^H NMR spectra of fermented juices are shown in S1 Fig. While PHA, HPLA, HIVA, GPC, and acetoin were initially detected as unidentified metabolites using SpinAssign, their identities were determined by a comparative spectral analysis with chemical standards.

As a spectral overview, signals of sugars such as Suc, Glc, and Fru predominated in the ^1^H NMR spectrum of control juice samples. MalA and CitA were the dominant organic acids. After fermentation, signal intensities of Glc, Fru, MalA, and FumA were fully or markedly reduced and those of LacA and HOAc were substantially increased. Although the decrease in pH almost plateaued within 3 days, the signal intensities of LacA and HOAc gradually increased over 3 weeks, indicating the sustained microbial activity under acidic condition. Intriguingly, the changes in the intensities of other metabolites depended on the lactic acid fermentation type (homo or hetero) and/or strain.

### Characterization by non-targeted multivariate analyses

**Fig 1:**
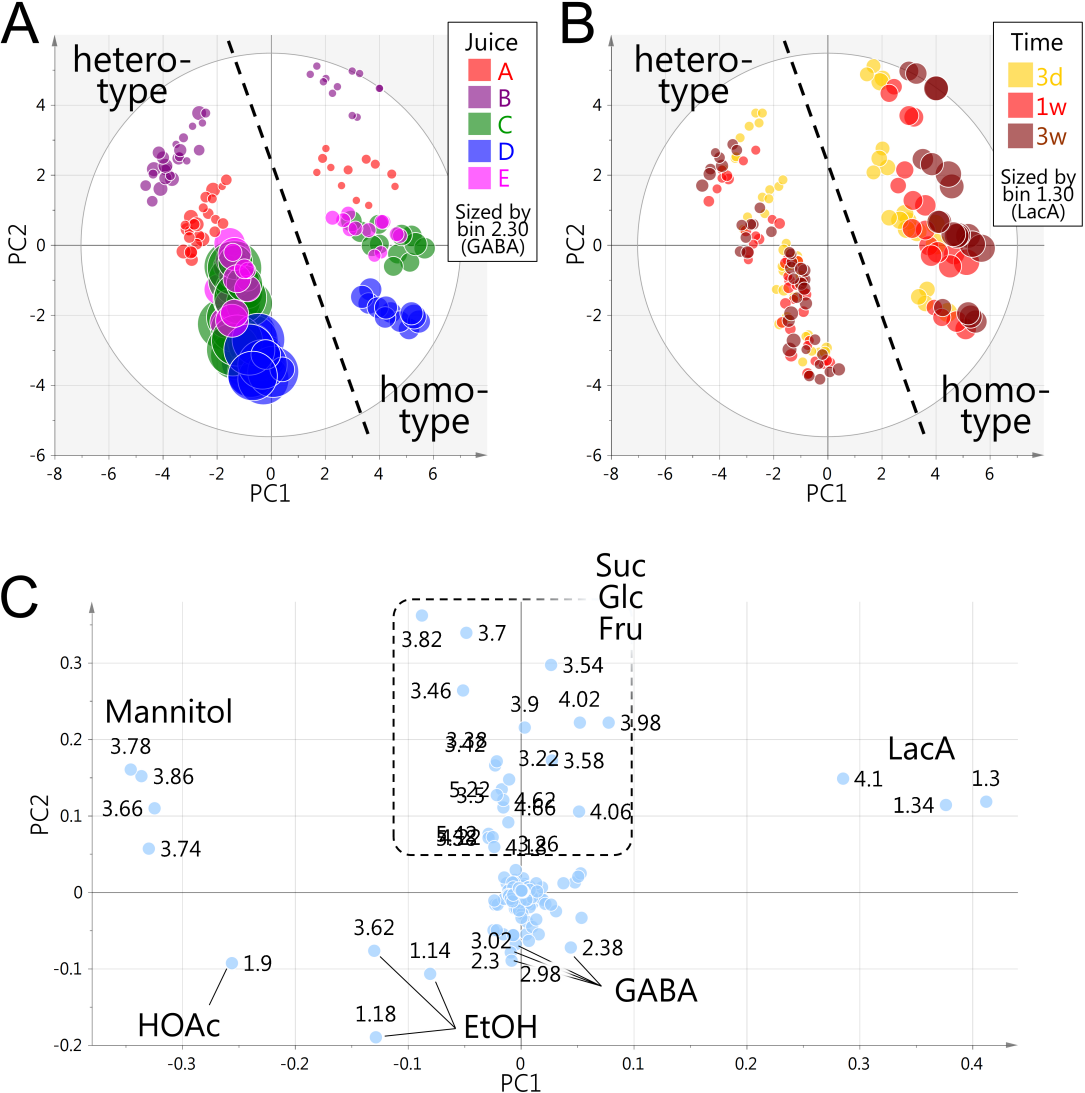
Non-targeted PCA of 180 fermented vegetable juice samples. First and second principal components (PC1 and PC2) represent 46.1% and 28.1% of the total variance, respectively. (A) Score plot color-coded according to juice. Symbols were sized by the signal derived from GABA at bin 2.30. (B) Score plot color-coded according to duration of fermentation. Symbols were sized by the signal derived from LacA at bin 1.30. (C) Loading plot. Labels represent the central chemical shifts (ppm) of integral buckets of 0.04-ppm width.

Initially, we analyzed 180 samples of prepared fermented juices with PCA, using a dataset generated by subdividing the ^1^H NMR spectra into 0.04-ppm width buckets. Score and loading plots are depicted in Fig 1. The first principal component (PC1, 46.1% of the total variance) showed a clear separation between homo- and hetero-fermentative LAB strains, indicating that this was a principal characteristic among samples rather than the difference in juices (A–E) or durations of fermentation (Fig 1A and 1B). In accordance with the theoretical metabolic pathways of homo- and hetero-fermentative LAB, loading of PC1 explained the class separation by LacA, EtOH, and HOAc (Fig 1C). A higher level of LacA was associated with homofermentative strains, which can produce two moles of LacA from one mole of Glc. By contrast, higher levels of EtOH and HOAc were associated with heterofermentative strains, which catabolize Glc not only to LacA but also to CO_2_, EtOH, and HOAc [8]. This may explain the difference in terminal pH between the fermentative types and can potentially impact the intensity of sourness of fermented foods. Mannitol also made a considerable contribution to this class separation along the PC1 axis even though it is not directly related to lactic acid fermentation. Whereas a substantial amount of mannitol accumulated in samples of heterofermentative strains, a substantial amount of Fru was consumed. In some heterofermentative *Lactobacillus* species, mannitol is directly converted from Fru by mannitol dehydrogenase [21]. In this reaction, Fru acts as an electron acceptor to regenerate NAD^+^ from NADH and thus contributes to maintaining the intracellular redox balance of heterofermentative strains [21]. Little or no mannitol was detected in samples of homofermentative strains, probably owing to Fru utilization through glycolysis. Mannitol production might influence the sensory quality of fermented foods owing to its sweet taste.

Class separations among the different juices (A–E) were evident along the PC2 axis, which accounted for 28.1% of the total variance (Fig 1A). The separation was explained primarily by signals derived from residual sugars, including Suc, Glc, and Fru, and by those of GABA, EtOH, and HOAc (Fig 1C). Specifically, fermented juices A and B had higher levels of residual sugars, whereas juices C–E contained higher levels of GABA and EtOH. Juices A and B contained higher initial levels of sugars, likely due to the presence of fruit juice. By contrast, juices C–E, which are 100% vegetable juices, had higher initial levels of amino acids. This may explain why juices C–E resulted in higher production levels of GABA, since GABA is produced by the decarboxylation of Glu. GABA production potentially has positive and negative impacts on the health-promoting and flavor qualities, respectively, because GABA is globally recognized as beneficial to health whereas it is produced with the consumption of Glu responsible for the *umami* flavor. The GABA-producing ability of LAB can be utilized in the production of high-value-added fermented foods [22]. Results from this analysis highlighted the heterofermentative *L*. *brevis* species, which accumulated high levels of GABA in agreement with a previous study [23].

Difference in incubation time had a relatively lower impact on the feature space of all 180 samples from the five juices. When PCA was carried out for each juice, incubation time was observed as a secondary characteristic following fermentation type. Factor loadings explained the sustained increase in LacA, HOAc, EtOH, and mannitol levels over the 3-week incubation period (data not shown). PCA score plots of juices C, D, and E highlighted the characteristics of certain strains within the homo- and hetero-fermentative groups. Within the heterofermentative strains, Lm1 and Lm12 formed a separate group from the other strains owing to their ability to rapidly degrade Suc. Within homofermentative strains, a class separation between Lm14 and the other strains showed that the initial level of Fru remained constant over 3 weeks in Lm14 samples, suggesting defective assimilation capacity of Fru in this strain.

Taken together, non-targeted PCA successfully discriminated between the fermentation characteristics of the sampled strains based on the type of lactic acid fermentation and the degradation capacity of predominant sugars. However, the data indicated the contributions of several dominant components, despite annotation of more than 50 metabolites. Most metabolites were present in low abundance and the dataset was inadequate for investigating the contribution of these minor metabolites. Thus, we employed an alternative method to prepare another dataset focused on the impact of minor metabolites.

### Advanced characterization by an ROIs-based approach

To investigate the impact of minor metabolites, we prepared a dataset based on manually specified integral regions for independent ^1^H NMR signals. This ROIs-based approach avoided the irrelevant effects arising from *Z*-score normalization of noise regions in the ^1^H NMR spectrum. In addition, it equalized the influence of major and minor metabolites, highlighting the contribution of the latter. The generated dataset contained 101 variables based on 76 integrals of 47 annotated metabolites and 25 unidentified signals. The integral regions and metabolite annotations are listed in Table 1.

**Table 1:** Manually specified integrals in ^1^H NMR spectrum used for ROIs-based analysis.

				range (ppm)	
#	annotation	abbrv.	assignment	start	end
1	2,3-butanediol		–CH_3_	1.14	1.12
2	2-hydroxyisovaleric acid	HIVA	–CH_3_	0.84	0.81
3	acetic acid	HOAc	–CH_3_	1.91	1.89
4	acetoin		>CH–CH_3_	1.38	1.35
5	acetoin		–(O =) C–CH_3_	2.22	2.20
6	acetoin		>CH–	4.44	4.43
7	alanine	Ala	Hβ	1.91	1.45
8	asparagine	Asn	Hβ	2.88	2.85
9	asparagine	Asn	Hα	2.97	2.94
10	aspartic acid	Asp	Hβ	2.70	2.69
11	aspartic acid	Asp	Hα	2.79	2.77
12	*NNN*-trimethylglycine		–CH_3_	3.29	3.29
13	chlorogenic acid	CGA	Phe–CH = CH–	6.40	6.38
14	chlorogenic acid	CGA	Phenolic H	6.96	6.95
15	chlorogenic acid	CGA	Phe–CH = CH–	7.66	7.65
16	choline		–CH_3_	3.19	3.18
17	citric acid	CitA	–CH_2_–	2.59	2.51
18	citric acid	CitA	–CH_2_–	2.65	2.61
19	dihydroxyacetone	DHA	–CH_2_–	4.41	4.40
20	ethanol	EtOH	–CH_3_	1.18	1.16
21	ethanol	EtOH	–CH_2_–	3.62	3.62
22	formic acid	ForA	–CH =	8.45	8.43
23	fructose	Fru	H-5 (β-form)	3.99	3.97
24	fructose	Fru	H-6 (β-form)	4.01	3.99
25	γ-aminobutyric acid	GABA	Hβ	1.89	1.85
26	γ-aminobutyric acid	GABA	Hα	2.31	2.26
27	γ-aminobutyric acid	GABA	Hγ	3.02	2.97
28	galactose	Gal	H-1 (α-form)	5.26	5.25
29	galacturonic acid	Galu	H-1 (α-form)	5.29	5.27
30	glucose	Glc	H-2 (α-form)	3.52	3.49
31	glucose	Glc	H-1 (β-form)	4.65	4.62
32	glucose	Glc	H-1 (α-form)	5.24	5.21
33	glutamic acid	Glu	Hγ	2.33	2.32
34	glycerophosphocholine	GPC	–CH_3_	3.21	3.20
35	histidine	His	imidazolic	7.17	7.09
36	histidine	His	imidazolic	8.04	7.88
37	isoleucine	Ile	Hδ	0.93	0.92
38	isoleucine	Ile	Hγ2	1.01	1.00
39	lactic acid	LacA	–CH_3_	1.34	1.29
40	lactic acid	LacA	>CH–	4.13	4.11
41	leucine	Leu	Hδ1, Hδ2	0.96	0.94
42	mannose	Man	H-1 (β-form)	4.89	4.88
43	mannose	Man	H-1 (α-form)	5.17	5.16
44	mannitol		H-1, H-6	3.66	3.66
45	mannitol		H-3, H-4	3.79	3.78
46	methanol	MeOH	–CH_3_	3.35	3.34
47	*myo*-inositol		H-5	3.27	3.26
48	ornithine	Orn	Hδ	3.06	3.04
49	pectin-like		multiple	5.12	5.04
50	phenylalanine	Phe	Hδ1, Hδ2	7.33	7.32
51	phenylalanine	Phe	Hε1, Hε2	7.44	7.39
52	phenyllactic acid	PLA	Hδ1, Hδ2	7.30	7.29
53	phenyllactic acid	PLA	Hε1, Hε2	7.36	7.35
54	*p*-hydroxyphenyllactic acid	HPLA	Hε1, Hε2	6.86	6.83
55	pyroglutamic acid	Glp	Hβ	1.99	1.98
56	pyroglutamic acid	Glp	Hγ	2.41	2.40
57	pyroglutamic acid	Glp	Hβ	2.50	2.45
58	pyroglutamic acid	Glp	Hα	4.18	4.14
59	rhamnose	Rha	H-6	1.27	1.25
60	*scyllo*-inositol		>CH–	3.34	3.33
61	sucrose	Suc	H-4ʹ	4.06	4.03
62	sucrose	Suc	H-3ʹ	4.23	4.19
63	sucrose	Suc	H-1	5.42	5.38
64	succinic acid	SucA	–CH_2_–	2.40	2.39
65	tartaric acid	TarA	>CH–	4.32	4.31
66	*tert*-butanol	*t*BuOH	–CH_3_	1.24	1.23
67	trehalose		H-1	5.19	5.17
68	tyrosine	Tyr	Hε1, Hε2	6.90	6.89
69	tyrosine	Tyr	Hδ1, Hδ2	7.20	7.18
70	tyramine		Hε1, Hε2	6.91	6.90
71	tyramine		Hδ1, Hδ2	7.22	7.21
72	uracil	Ura	–CH = CH–NH–	5.80	5.78
73	uracil	Ura	–CH = CH–NH–	7.54	7.51
74	uridine monophosphate	UMP	–CH = CH–NH–	6.00	5.95
75	valine	Val	Hγ1	0.98	0.97
76	valine	Val	Hγ2	1.03	1.02
77	U_0.78			0.79	0.76
78	U_1.50			1.52	1.49
79	U_1.98			1.98	1.97
80	U_2.20			2.20	2.19
81	U_3.03			3.04	3.03
82	U_3.10			3.10	3.09
83	U_4.29			4.30	4.29
84	U_4.36			4.36	4.36
85	U_4.37			4.38	4.36
86	U_4.96			4.97	4.95
87	U_5.31			5.32	5.30
88	U_5.42			5.43	5.42
89	U_5.61			5.63	5.60
90	U_5.67			5.68	5.66
91	U_6.13			6.14	6.12
92	U_6.35			6.36	6.35
93	U_6.42			6.44	6.41
94	U_6.92			6.92	6.91
95	U_8.19			8.19	8.18
96	U_8.20			8.21	8.20
97	U_8.23			8.24	8.23
98	U_8.26			8.26	8.25
99	U_8.57			8.59	8.56
100	U_8.83			8.85	8.81
101	U_9.11			9.12	9.11
–	(Internal standard)	DSS	–CH_2_–	0.67	0.58

**Fig 2:**
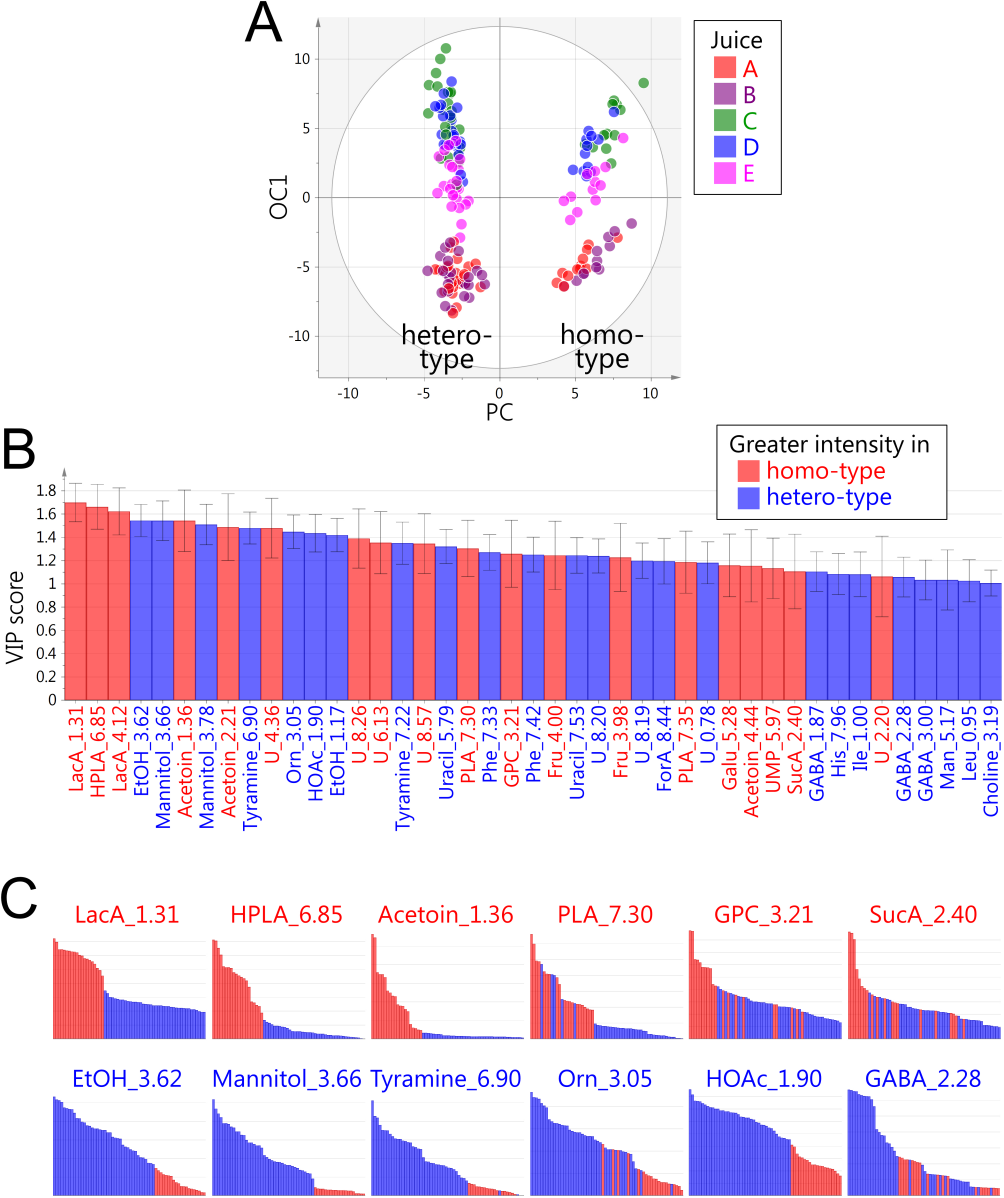
ROIs-based OPLS-DA of homo- and hetero-fermentative strains. (A) Score plot color-coded according to juice. Predictive component (PC) and first orthogonal component (OC1) explained 19.9% and 24.6% of the total variance, respectively. (B) VIP scores. Metabolites with scores of >1.0 are shown in descending order. Red and blue bars show higher levels in samples obtained from homo- and hetero-fermentative strain samples, respectively. Metabolites and chemical shifts are as shown in Table 1. (C) Intact integral values of annotated metabolites responsible for separations. The values are of samples fermented for 3 weeks without *Z*-score transformation. Colors represent fermentation type as in panel B.

Using the generated dataset, OPLS-DA was carried out to investigate the difference between the metabolite profiles of the two fermentation types. The determinant coefficient (R^2^) and cross-validation determination coefficient (Q^2^) values were 0.960 and 0.953, respectively. Homo- and hetero-fermentative strains were clearly separated in the score plot (Fig 2A). Variable importance in projection (VIP) identified the metabolites responsible for the class separation (Fig 2B). Significant contributions of LacA, HOAc, EtOH, and mannitol agreed with the non-targeted analysis (S2 Fig), validating the model constructed using this approach. In addition to these major components, various low-abundance metabolites were strongly correlated with the lactic acid fermentation type. The metabolites and their possible impact on the quality of fermented foods are described below.

Metabolites associated with the homofermentative strains—LacA, HPLA, acetoin, PLA, GPC, and SucA (Fig 2B)—were produced at higher levels by the homo- than the hetero-fermentative strains (Fig 2C). In the homofermentative species *L*. *plantarum*, acetoin is synthesized from pyruvic acid (PyrA) through α-acetolactic acid by acetolactate synthase and α-acetolactate decarboxylase (EC: 2.2.1.6 and 4.1.1.5, respectively) [24]. Acetoin and diacetyl (2,3-butanedione) are aromatic compounds produced by LAB and contribute to a buttery flavor [4]. As demonstrated by a previous study of a *L*. *plantarum* strain, acetoin production from pyruvate is also biologically important for acid tolerance as the decarboxylation reaction consumes protons, contributing to intracellular pH homeostasis [25]. Furthermore, acetoin production is associated with NAD^+^ regeneration, which is required for glycolysis [24], suggesting a role in redox balance. PLA and HPLA are aromatic compounds that impart floral and chemical flavors, respectively, and are present in fermented dairy products [5]. PLA and HPLA are converted by a transaminase-catalyzed reaction from Phe and Tyr through phenylpyruvic acid and *p*-hydroxyphenylpyruvic acid, respectively [5]. GPC is a moiety of phosphatidylcholine and can be formed by its deacylation. As lipase activity has been reported in LAB [26], the increase in glycerol 3-phosphate levels in the samples obtained from homofermentative strains might suggest active lipid degradation during fermentation. SucA, a component of the tricarboxylic acid (TCA) pathway, contributes to the flavor and *umami* taste. Some *Lactobacillus* strains can produce SucA from CitA via the incomplete TCA pathway, which is associated with NAD^+^ regeneration [27]. The fact that Lm14 (*L*. *pentosus*) accumulated a particularly high level of SucA and consumed CitA in our study agrees with these previous reports.

The samples obtained from heterofermentative strains were characterized by the production of higher levels of mannitol, EtOH, HOAc, Orn, tyramine, and GABA (Fig 2C). Orn is a component of the urea cycle and is produced either directly from Arg by Arg-urease or through citrulline by Arg-deiminase. Previous studies have reported that the latter pathway is detected in LAB strains obtained from wine and induced at low pH values [28, 29]. This may suggest that Orn production is involved in pH tolerance. GABA and tyramine are decarboxylation products of Glu and Tyr, respectively. The greater production of GABA and tyramine in heterofermentative strains contrasts with the fact that homofermentative strains converted Tyr and Phe by transamination. The decarboxylation of amino acids involves the elimination of protons, contributing to intracellular pH homeostasis and ATP production [30]. Although amino acid decarboxylation can produce beneficial compounds, such as GABA from Glu, the products of other amino acids (including tyramine) are classified as undesirable biogenic amines, which must be taken into account for food safety [31].

### Strain-dependent characteristics of *L*. *brevis* strains

**Fig 3:**
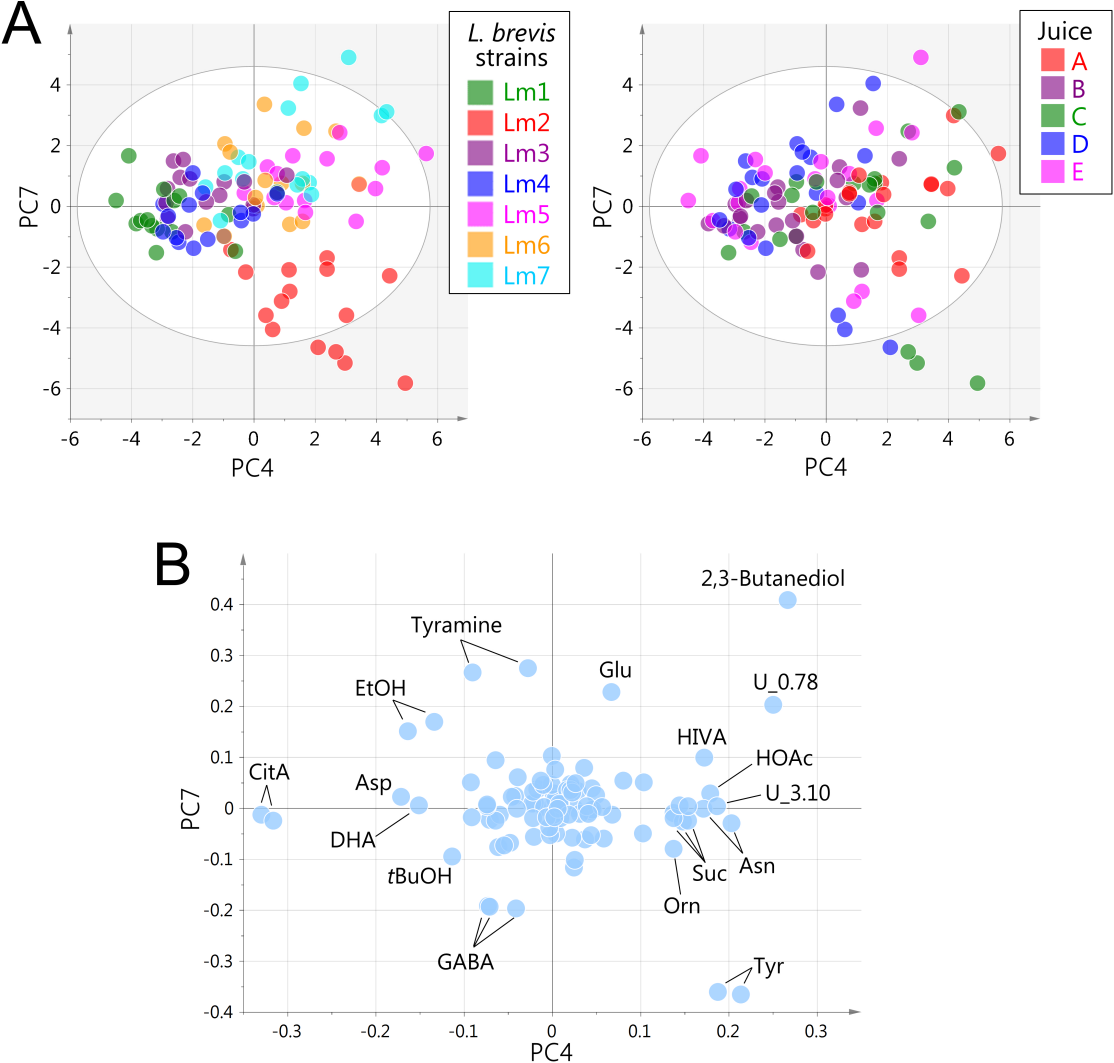
ROIs-based analysis of fermentation characteristics of *L*. *brevis* strains. The PC4–PC7 plane (7.2% and 4.2% of the total variance, respectively) is shown, as it represented the differences among the samples the most. (A) Score plot color-coded according to seven *L*. *brevis* strains (left) or five vegetable juices (right). (B) Loading plot.

To further evaluate the discrimination capacity of NMR metabolomics, we investigated strain-dependent characteristics within the same species. PCA on the 105 samples across seven strains of *L*. *brevis* that were assessed as active GABA producers in vegetable juice, were performed. This generated 10 principal components with a cumulative 90.2% of the total variance. The first three principal components primarily represented class separations among juices A–E rather than the strains (S3 Fig). Of the remaining principal components, the score plot of the PC4–PC7 plane reflected the differences among the *L*. *brevis* strains (Fig 3A). PC4 and PC7 explained 7.1% and 4.2% of the total variance, respectively. The loadings of PC4 and PC7 indicated that various metabolites described this plane (Fig 3B), primarily including CitA, Tyr, tyramine, Glu, GABA, 2,3-butanediol, and an unidentified signal at 0.78 ppm (U_0.78). Although contribution rates of these PCs were relatively small, we confirmed that the highlighted compounds were responsible for strain-dependent characteristics by performing PCA on each juice.

**Fig 4:**
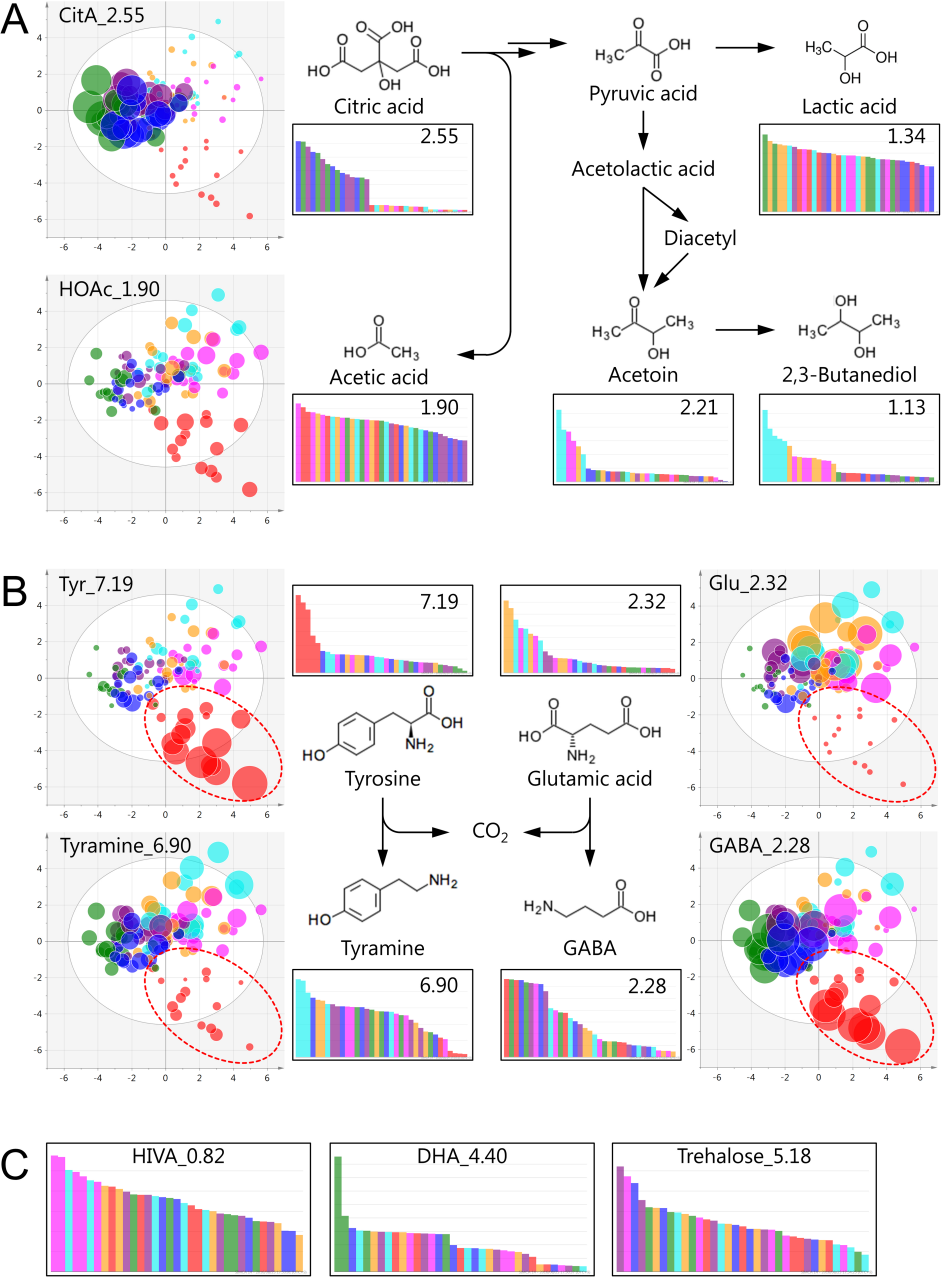
Levels and suggested pathways of metabolites responsible for differentiating tested *L*. *brevis* strains. The same PC4–PC7 plane shown in Fig 3 is used. Symbol size represents the integral values of the metabolites. Bar graphs show the integral values of metabolites in samples fermented for 3 weeks. Color coding corresponds to Fig 3. The metabolic pathways shown are from previous studies and the KEGG pathway database (http://www.genome.jp/kegg/pathway). (A) CitA fermentation pathway. (B) Tyr and Glu conversion to tyramine and GABA, respectively. The broken-line ellipse represents the separated class of strain Lm2. (C) Other characteristic metabolites that varied among strains and/or juices.

Along the PC4 axis, a tendency towards class separation among groups Lm1, Lm3, and Lm4, and groups Lm2, Lm5, Lm6, and Lm7, was observed (Fig 3A). CitA, 2,3-butanediol, and U_0.78 explained this separation (Fig 3B). The Lm2 group consumed almost all the CitA after 3 days whereas samples fermented by the Lm1 group contained a large quantity of residual CitA after 3 weeks (Fig 4A). CitA is catabolized to PyrA through oxaloacetic acid, which is associated with the consumption of H^+^ and formation of HOAc [24, 32]. LacA level was not correlated with CitA consumption. The fact that relatively higher levels of HOAc and 2,3-butanediol (and acetoin, in part) were detected in the Lm2 group (Fig 4A), might suggest that CitA was not catabolized to LacA but to 2,3-butanediol via acetolactic acid and diacetyl. The strain-dependent activity observed in CitA fermentation might be important for flavor control of fermented foods, since acetoin and diacetyl are aromatic compounds preferred for cheese and yogurt, but undesirable for rice wine (Japanese *sake*). By contrast, the downstream metabolite, 2,3-butanediol, is an odorless compound.

The PC7 axis revealed that Lm2 had different levels of Tyr, Glu, tyramine, and GABA compared to the other *L*. *brevis* strains (Fig 4B). Intriguingly, juices fermented by Lm2 retained a high level of Tyr and a low level of tyramine for 3 weeks, indicating the strain-dependent activity of Tyr–tyramine conversion. Furthermore, the data also characterized Glu–GABA conversion in Lm2. Fermenting juices C, D, and E with Lm2 resulted in a higher level of GABA compared to fermenting with other strains (Fig 4B). This might be because Lm2 depends on the decarboxylation of Glu, rather than Tyr, for maintaining intracellular pH. Consequently, the analysis successfully discriminated strain-dependent fermentation characteristics among *L*. *brevis* strains and identified a potential strain that can produce GABA without tyramine accumulation, which may be highly desirable for producing value-added fermented foods.

The PCA data also suggested that other metabolites are unique to certain *L*. *brevis* strains and to combinations of certain juices and strains. HIVA was detected at a high level when juices were fermented by strain Lm5 (Fig 4C). Production of HIVA in *L*. *brevis* has not been reported previously. However, in *Lactococcus lactis*, HIVA is a transamination product of Val [33]. This was in agreement with our study where the Lm5 sample showed a lower level of Val than other strains. Moreover, HIVA has been reported as a potential metabolic marker of vaginal bacterial diversity [34] and an antifungal compound [35]. The NMR signal of DHA (also known as glyceron) was present only in juice B fermented by Lm1 (Fig 4C). Although DHA is an intermediate of glycerol fermentation in *L*. *brevis* [36], the reason for this specific case of DHA production by Lm1 is unclear. Trehalose accumulation was the highest in juice B fermented by Lm3, followed by Lm5 and Lm4 (Fig 4C). Trehalose plays a key role in stress tolerance in LAB and its high water retention capability enhances cell survival under stress conditions, such as freezing and drying [37].

## Conclusions

This study demonstrated the applicability of NMR-based metabolomics for the rapid discrimination of fermentation characteristics of *Lactobacillus* strains. The use of fermented vegetable juice provided comprehensive information on metabolite changes under conditions similar to those of practical fermented food production. Metabolite profiles obtained by the ROIs-based approach enabled us to assess the contribution of low-abundance and unannotated metabolites. The analyses illuminated the differences among various metabolites involved in key metabolic pathways of LAB occurring in fermented food production, such as lactic acid fermentation from carbohydrates, consumption of MalA and CitA, decarboxylation and transamination of amino acids, and production of mannitol, SucA, and acetoin. Taken together, we propose that discrimination by NMR metabolomics is a high-throughput method for screening unique *Lactobacillus* strains from a large set of samples. This method may also be applicable to LAB strains selected from other genera such as *Lactococcus*, *Leuconostoc*, *Pediococcus*, and *Streptococcus*. Moreover, real-time NMR metabolomics [15, 38] can provide further information on dynamic metabolic changes in selected strains and facilitate the application of their fermentation characteristics to fermented food production.

## Supporting information

S1 Fig Representative ^1^H NMR spectra of fermented vegetable juices. (A) juice A fermented with Lm5. (B) juice B with Lm1. (C) juice C with Lm23. (D) juice D with Lm2. (E) juice E with Lm14. Numerical labels represent signals used for ROIs-based analysis, corresponding to those in Table 1.
(PDF)

S2 Fig Non-targeted OPLS-DA of homo- and hetero-lactic fermentative strains. The model was evaluated by leave-one-out cross validation, providing determinant coefficient (R^2^) and cross-validation determination coefficient (Q^2^) of 0.965 and 0.951, respectively. (A) Score plot color-coded according to juices. Predictive component (PC) and first orthogonal component (OC1) represent 43.1% and 29.9% of the total variance, respectively. (B) VIP scores. Metabolites with a score >1.0 are shown in descending order. Red and blue bars show higher levels in the samples of homo- and hetero-fermentative strains. Variable labels represent central chemical shifts of each bin (0.04-ppm width).
(PDF)

S3 Fig Projections of ROIs-based PCA data for tested *L*. *brevis* strains. Color code corresponds to Fig 3. The ten principal components explained the total variance as follows: PC1, 38.1%; PC2, 12.7%; PC3, 9.4%; PC4, 7.1%; PC5, 6.4%; PC6, 5.0%; PC7, 4.5%; PC8, 3.6%; PC9, 1.9%; PC10, 1.6%.
(PDF)

S1 Table Nutritional composition of vegetable juice products used in this study. (PDF)

## References

[1] Tamang JP, Watanabe K, Holzapfel WH. Review: Diversity of microorganisms in global fermented foods and beverages. Front Microbiol. 2016;7:377 doi: 10.3389/fmicb.2016.00377 27047484 10.3389/fmicb.2016.00377 PMC4805592

[2] Steinkraus KH. Classification of fermented foods: Worldwide review of household fermentation techniques. Food Control. 1997;8(5–6):311–317. doi: 10.1016/S0956-7135(97)00050-9

[3] Tamang JP, Shin DH, Jung SJ, Chae SW. Functional properties of microorganisms in fermented foods. Front Microbiol. 2016;7:578 doi: 10.3389/fmicb.2016.00578 27199913 10.3389/fmicb.2016.00578 PMC4844621

[4] Smit G, Smit BA, Engels WJ. Flavour formation by lactic acid bacteria and biochemical flavour profiling of cheese products. FEMS Microbiol Rev. 2005;29(3):591–610. doi: 10.1016/j.femsre.2005.04.002 15935512 10.1016/j.femsre.2005.04.002

[5] Smid EJ, Kleerebezem M. Production of aroma compounds in lactic fermentations. Annu Rev Food Sci Technol. 2014;5:313–326. doi: 10.1146/annurev-food-030713-092339 24580073 10.1146/annurev-food-030713-092339

[6] Ganzle MG, Vermeulen N, Vogel RF. Carbohydrate, peptide and lipid metabolism of lactic acid bacteria in sourdough. Food Microbiol. 2007;24(2):128–138. doi: 10.1016/j.fm.2006.07.006 17008155 10.1016/j.fm.2006.07.006

[7] Mozzi F, Ortiz ME, Bleckwedel J, De Vuyst L, Pescuma M. Metabolomics as a tool for the comprehensive understanding of fermented and functional foods with lactic acid bacteria. Food Res Int. 2013;54(1):1152–1161. doi: 10.1016/j.foodres.2012.11.010

[8] Ravyts F, De Vuyst L, Leroy F. Bacterial diversity and functionalities in food fermentations. Eng Life Sci. 2012;12(4):356–367. doi: 10.1002/elsc.201100119

[9] Li H, Cao Y. Lactic acid bacterial cell factories for gamma-aminobutyric acid. Amino Acids. 2010;39(5):1107–1116. doi: 10.1007/s00726-010-0582-7 20364279 10.1007/s00726-010-0582-7

[10] Hong YS. NMR-based metabolomics in wine science. Magn Reson Chem. 2011;49 Suppl 1(S1):S13–21. doi: 10.1002/mrc.2832 22290704 10.1002/mrc.2832

[11] Settachaimongkon S, Nout MJ, Antunes Fernandes EC, Hettinga KA, Vervoort JM, van Hooijdonk TC, et al Influence of different proteolytic strains of *Streptococcus thermophilus* in co-culture with *Lactobacillus delbrueckii* subsp. *bulgaricus* on the metabolite profile of set-yoghurt. Int J Food Microbiol. 2014;177:29–36. doi: 10.1016/j.ijfoodmicro.2014.02.008 24598513 10.1016/j.ijfoodmicro.2014.02.008

[12] Lu Y, Hu F, Miyakawa T, Tanokura M. Complex mixture analysis of organic compounds in yogurt by NMRspectroscopy. Metabolites. 2016;6(2):19 doi: 10.3390/metabo6020019 27322339 10.3390/metabo6020019 PMC4931550

[13] Ochi H, Naito H, Iwatsuki K, Bamba T, Fukusaki E. Metabolomics-based component profiling of hard and semi-hard natural cheeses with gas chromatography/time-of-flight-mass spectrometry, and its application to sensory predictive modeling. J Biosci Bioeng. 2012;113(6):751–758. doi: 10.1016/j.jbiosc.2012.02.006 22386562 10.1016/j.jbiosc.2012.02.006

[14] Palama TL, Canard I, Rautureau GJ, Mirande C, Chatellier S, Elena-Herrmann B. Identification of bacterial species by untargeted NMR spectroscopy of the exo-metabolome. Analyst. 2016;141(15):4558–4561. doi: 10.1039/c6an00393a 27349704 10.1039/c6an00393a

[15] Fukuda S, Nakanishi Y, Chikayama E, Ohno H, Hino T, Kikuchi J. Evaluation and characterization of bacterial metabolic dynamics with a novel profiling technique, real-time metabolotyping. PLoS One. 2009;4(3):e4893 doi: 10.1371/journal.pone.0004893 19287504 10.1371/journal.pone.0004893 PMC2654759

[16] Wegkamp A, Teusink B, de Vos WM, Smid EJ. Development of a minimal growth medium for *Lactobacillus plantarum*. Lett Appl Microbiol. 2010;50(1):57–64. doi: 10.1111/j.1472-765X.2009.02752.x 19874488 10.1111/j.1472-765X.2009.02752.x

[17] Bringel F, Quenee P, Tailliez P. Polyphasic investigation of the diversity within *Lactobacillus plantarum* related strains revealed two *L*. *plantarum* subgroups. Syst Appl Microbiol. 2001;24(4):561–571. doi: 10.1078/0723-2020-00061 11876364 10.1078/0723-2020-00061

[18] Rivas-Ubach A, Perez-Trujillo M, Sardans J, Gargallo-Garriga A, Parella T, Penuelas J. Ecometabolomics: Optimized NMR-based method. Methods in Ecology and Evolution. 2013;4(5):464–473. doi: 10.1111/2041-210x.12028

[19] Tomita S, Nemoto T, Matsuo Y, Shoji T, Tanaka F, Nakagawa H, et al A NMR-based, non-targeted multistep metabolic profiling revealed l-rhamnitol as a metabolite that characterised apples from different geographic origins. Food Chem. 2015;174:163–172. doi: 10.1016/j.foodchem.2014.11.028 25529666 10.1016/j.foodchem.2014.11.028

[20] Lewis IA, Schommer SC, Markley JL. Rnmr: Open source software for identifying and quantifying metabolites in NMR spectra. Magn Reson Chem. 2009;47 Suppl 1(S1):S123–126. doi: 10.1002/mrc.2526 19821464 10.1002/mrc.2526 PMC2798074

[21] Wisselink HW, Weusthuis RA, Eggink G, Hugenholtz J, Grobben GJ. Mannitol production by lactic acid bacteria: A review. Int Dairy J. 2002;12(2–3):151–161. doi: 10.1016/S0958-6946(01)00153-4

[22] Hagi T, Kobayashi M, Nomura M. Metabolome analysis of milk fermented by gamma-aminobutyric acid-producing lactococcus lactis. J Dairy Sci. 2016;99(2):994–1001. doi: 10.3168/jds.2015-9945 26686724 10.3168/jds.2015-9945

[23] Wu Q, Shah NP. High gamma-aminobutyric acid production from lactic acid bacteria: Emphasis on *Lactobacillus brevis* as a functional dairy starter. Crit Rev Food Sci Nutr. 2016:0. doi: 10.1080/10408398.2016.1147418 26980301 10.1080/10408398.2016.1147418

[24] Ferain T, Schanck AN, Delcour J. ^13^C nuclear magnetic resonance analysis of glucose and citrate end products in an *ldhL*-*ldhD* double-knockout strain of *Lactobacillus plantarum*. J Bacteriol. 1996;178(24):7311–7315. 8955418 10.1128/jb.178.24.7311-7315.1996 PMC178649

[25] Tsau JL, Guffanti AA, Montville TJ. Conversion of pyruvate to acetoin helps to maintain pH homeostasis in *Lactobacillus plantarum*. Appl Environ Microbiol. 1992;58(3):891–894. 16348677 10.1128/aem.58.3.891-894.1992 PMC195350

[26] Meyers SA, Cuppett SL, Hutkins RW. Lipase production by lactic acid bacteria and activity on butter oil. Food Microbiol. 1996;13(5):383–389. doi: 10.1006/fmic.1996.0044

[27] Kaneuchi C, Seki M, Komagata K. Production of succinic acid from citric acid and related acids by *Lactobacillus* strains. Appl Environ Microbiol. 1988;54(12):3053–3056. 16347795 10.1128/aem.54.12.3053-3056.1988 PMC204426

[28] Liu SQ, Pritchard GG, Hardman MJ, Pilone GJ. Arginine catabolism in wine lactic acid bacteria: Is it via the arginine deiminase pathway or the arginase-urease pathway? J Appl Bacteriol. 1996;81(5):486–492.

[29] Vrancken G, Rimaux T, Weckx S, De Vuyst L, Leroy F. Environmental pH determines citrulline and ornithine release through the arginine deiminase pathway in *Lactobacillus fermentum* IMDO 130101. Int J Food Microbiol. 2009;135(3):216–222. doi: 10.1016/j.ijfoodmicro.2009.07.035 19732985 10.1016/j.ijfoodmicro.2009.07.035

[30] Higuchi T, Hayashi H, Abe K. Exchange of glutamate and gamma-aminobutyrate in a *Lactobacillus* strain. J Bacteriol. 1997;179(10):3362–3364. 9150237 10.1128/jb.179.10.3362-3364.1997 PMC179120

[31] Shalaby AR. Significance of biogenic amines to food safety and human health. Food Res Int. 1996;29(7):675–690. doi: 10.1016/S0963-9969(96)00066-X

[32] Hickey MW, Hillier AJ, Jago GR. Metabolism of pyruvate and citrate in lactobacilli. Aust J Biol Sci. 1983;36(5–6):487–496. 6426447 10.1071/bi9830487

[33] Novak L, Loubiere P. The metabolic network of *Lactococcus lactis*: Distribution of ^14^C-labeled substrates between catabolic and anabolic pathways. J Bacteriol. 2000;182(4):1136–1143. doi: 10.1128/jb.182.4.1136–1143.2000 10648541 10.1128/jb.182.4.1136-1143.2000 PMC94391

[34] McMillan A, Rulisa S, Sumarah M, Macklaim JM, Renaud J, Bisanz JE, et al A multi-platform metabolomics approach identifies highly specific biomarkers of bacterial diversity in the vagina of pregnant and non-pregnant women. Sci Rep. 2015;5:14174 doi: 10.1038/srep14174 26387596 10.1038/srep14174 PMC4585667

[35] Honore AH, Aunsbjerg SD, Ebrahimi P, Thorsen M, Benfeldt C, Knochel S, et al Metabolic footprinting for investigation of antifungal properties of *Lactobacillus paracasei*. Anal Bioanal Chem. 2016;408(1):83–96. doi: 10.1007/s00216-015-9103-6 26573172 10.1007/s00216-015-9103-6

[36] Vivek N, Pandey A, Binod P. Biological valorization of pure and crude glycerol into 1,3-propanediol using a novel isolate *Lactobacillus brevis* N1E9.3.3. Bioresour Technol. 2016;213:222–230. doi: 10.1016/j.biortech.2016.02.020 26920628 10.1016/j.biortech.2016.02.020

[37] Zayed G, Roos YH. Influence of trehalose and moisture content on survival of *Lactobacillus salivarius* subjected to freeze-drying and storage. Process Biochem. 2004;39(9):1081–1086. doi: 10.1016/S0032-9592(03)00222-X

[38] Ebrahimi P, Larsen FH, Jensen HM, Vogensen FK, Engelsen SB. Real-time metabolomic analysis of lactic acid bacteria as monitored by in vitro NMR and chemometrics. Metabolomics. 2016;12(4):77 doi: 10.1007/s11306-016-0996-7

