# Supplementary figures and images for: Rapid discrimination of strain-dependent fermentation characteristics among Lactobacillus strains by NMR-based metabolomics of fermented vegetable juice
Source: PLoS One. 2017 Jul 31;12(7):e0182229. doi: 10.1371/journal.pone.0182229 (PMC5536307; doi:10.1371/journal.pone.0182229)

A

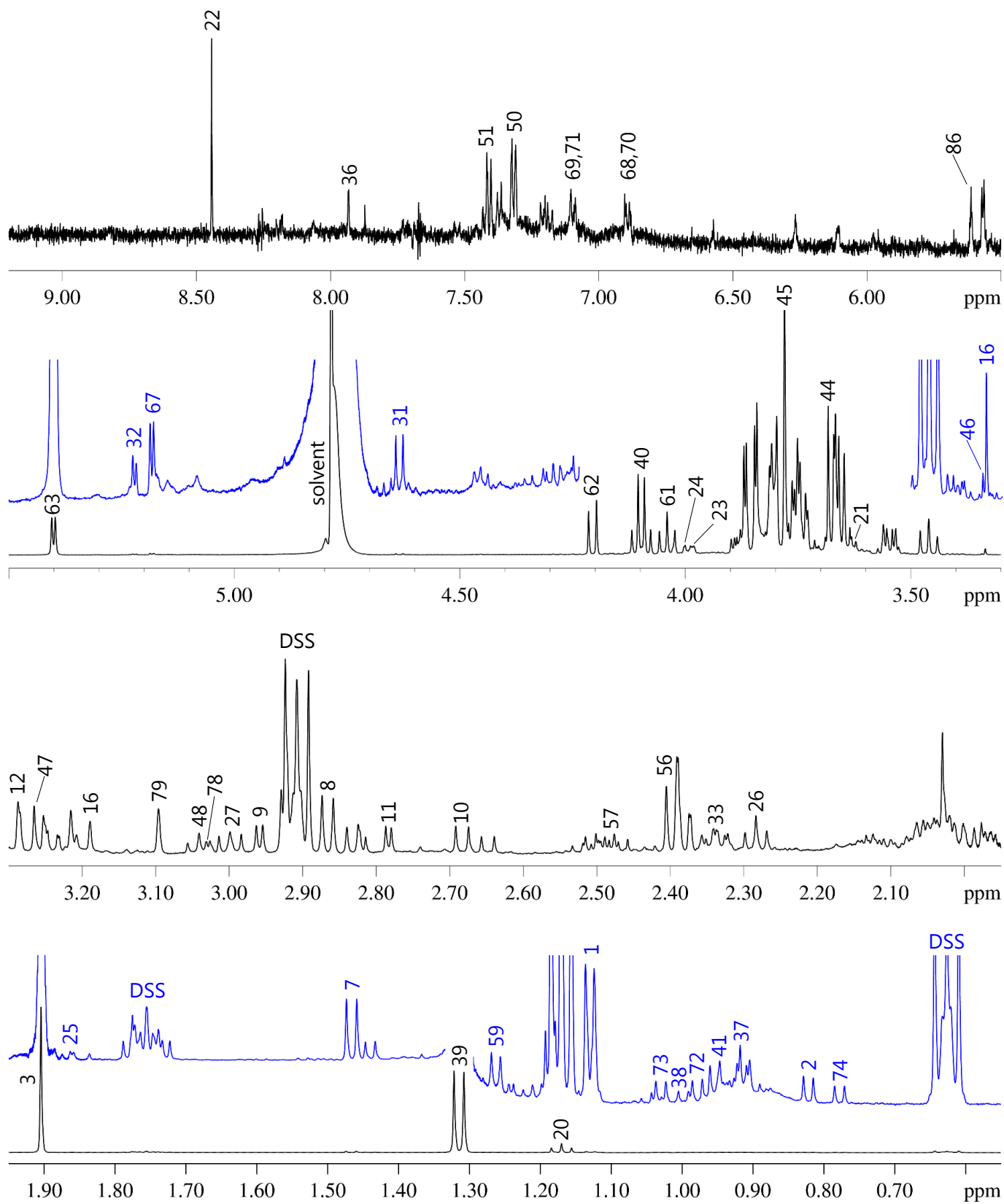

B

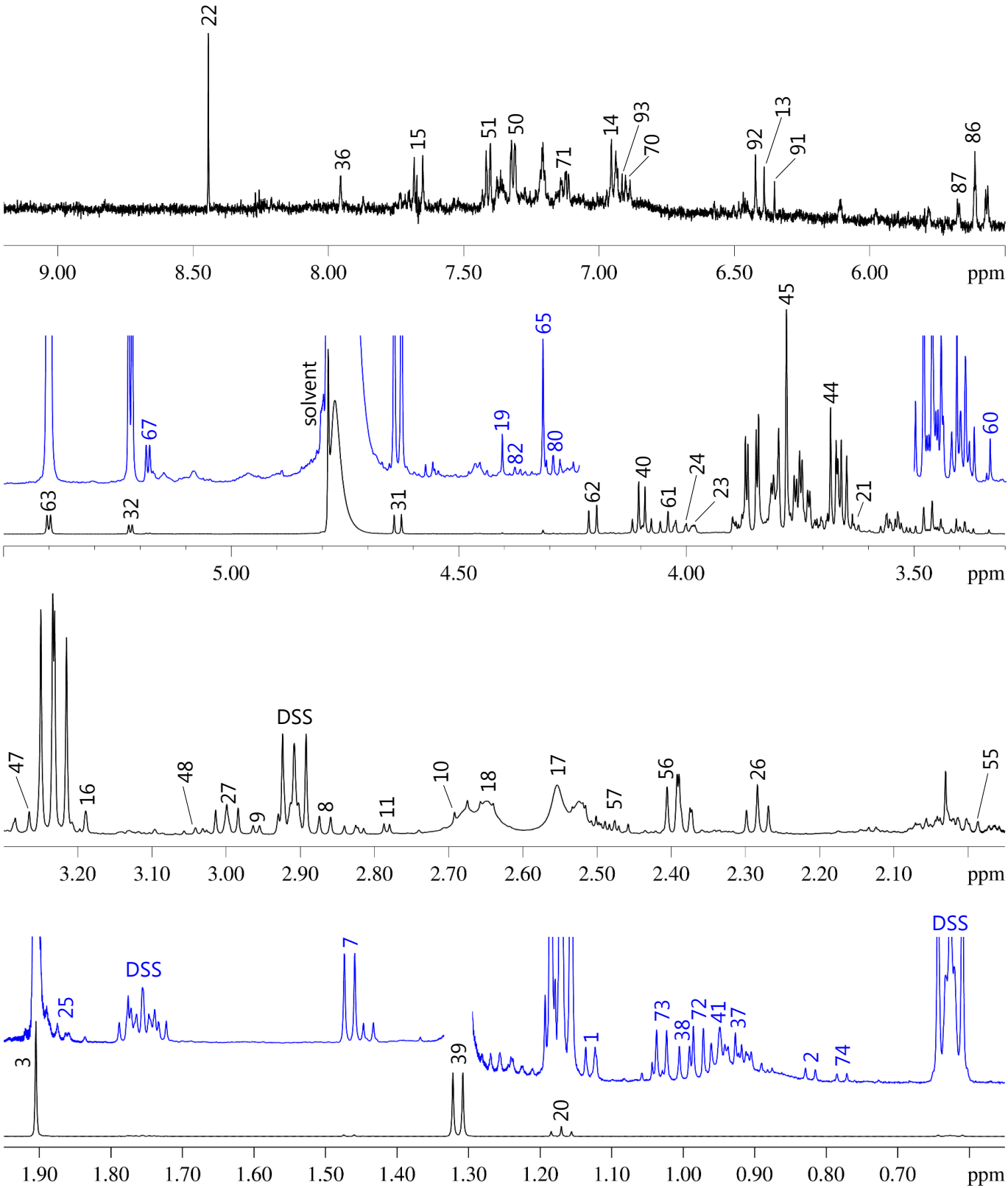

C

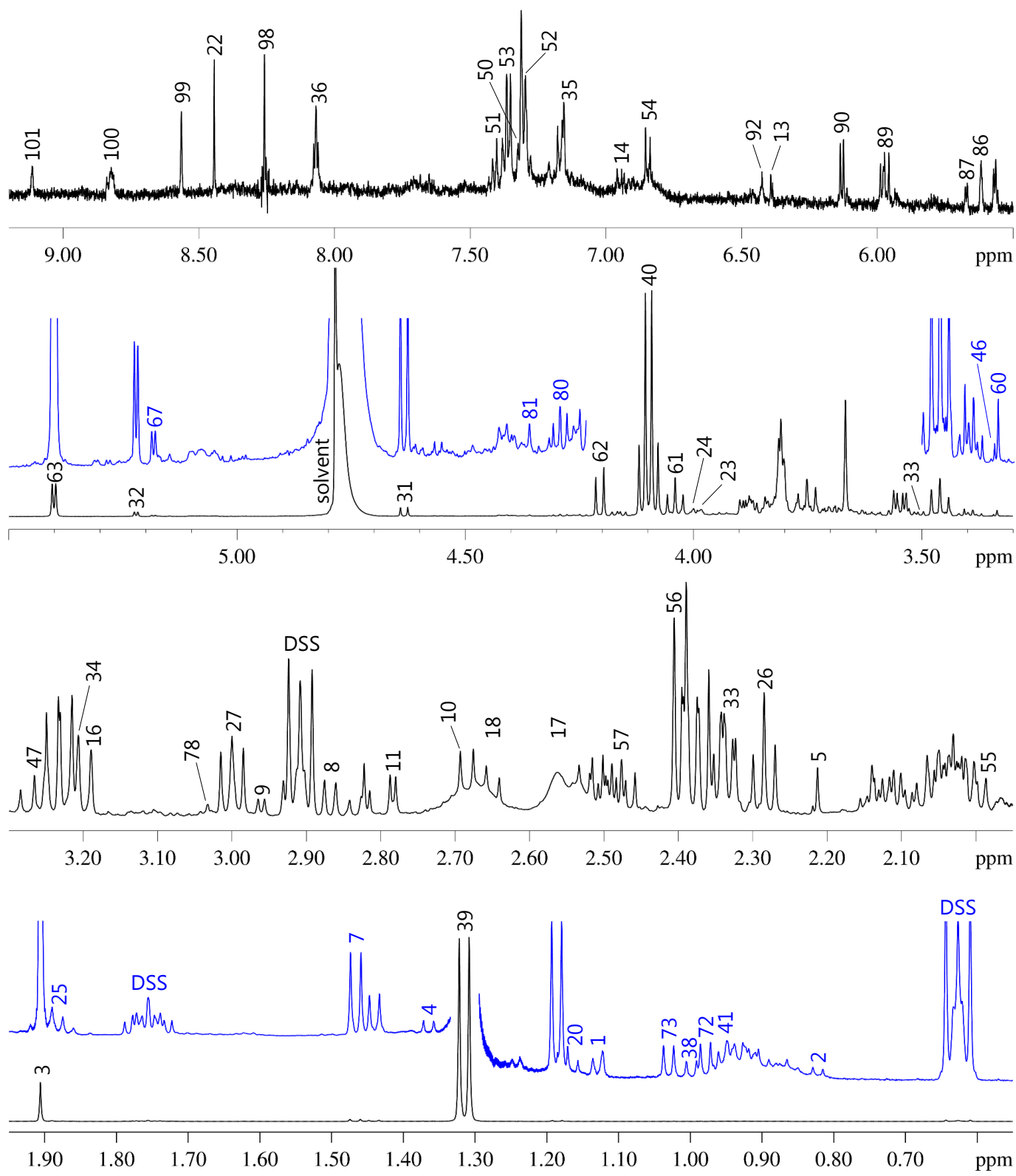

D

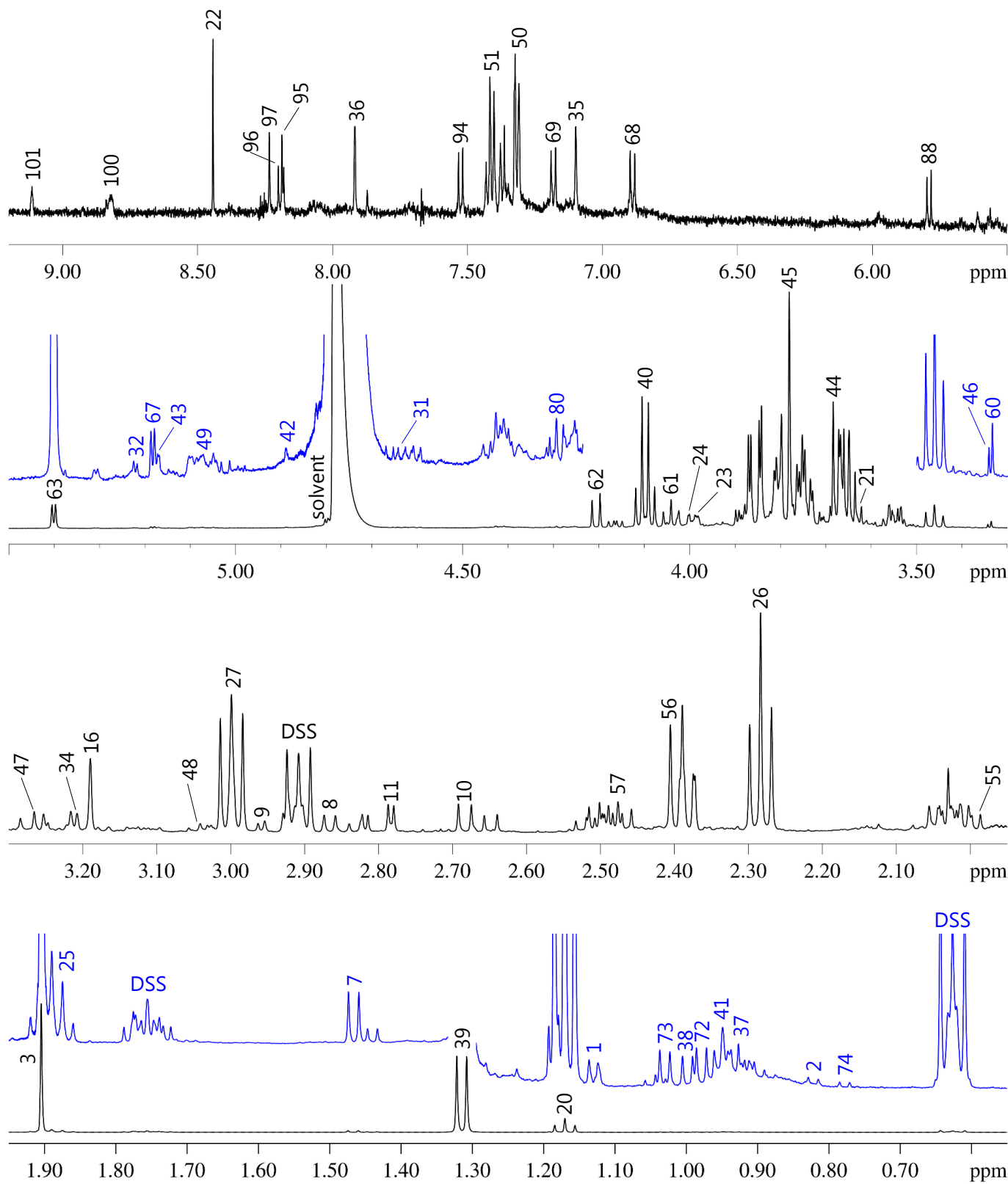

E

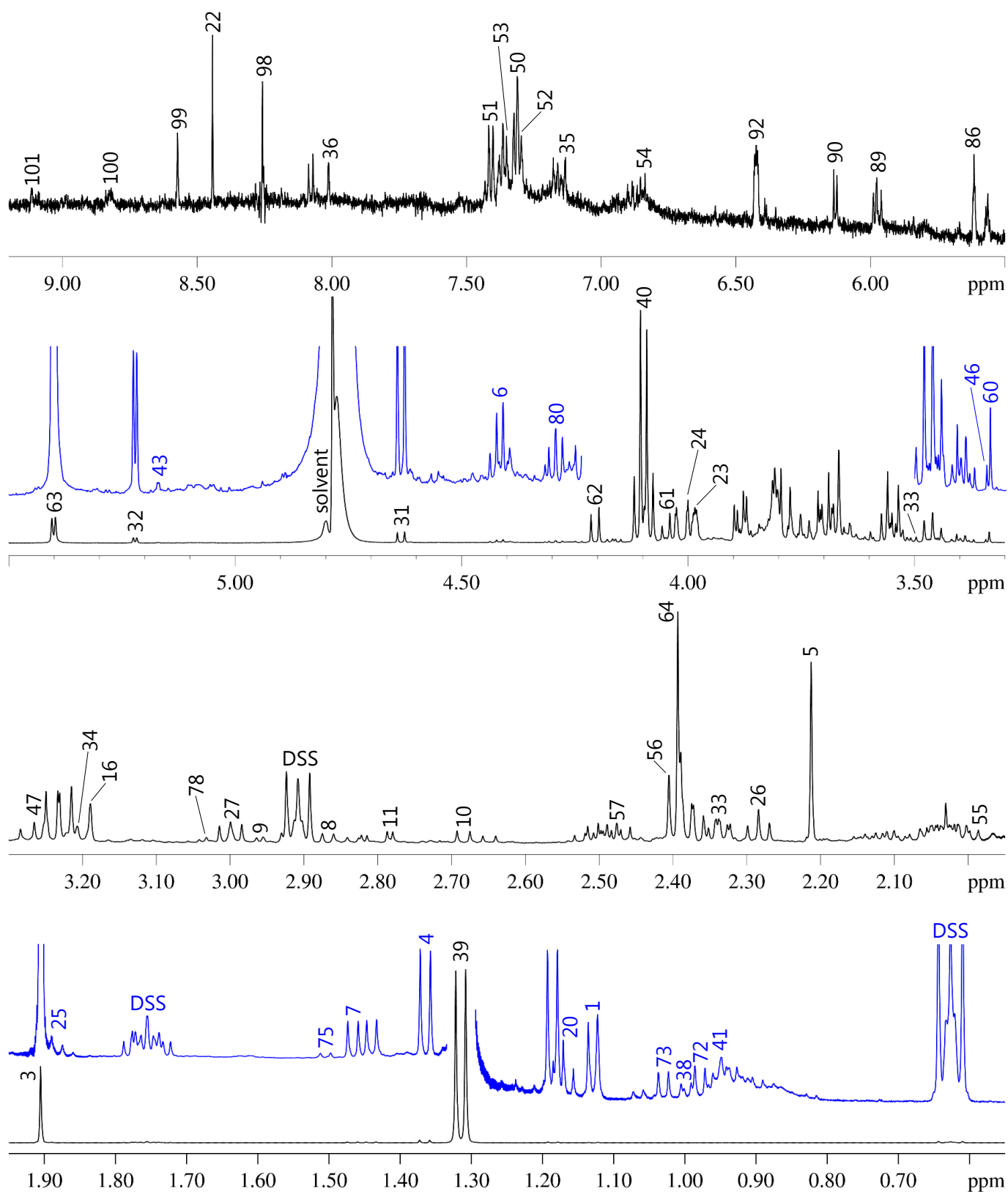

Supplement: S1 Fig — (A) juice A fermented with Lm5. (B) juice B with Lm1. (C) juice C with Lm23. (D) juice D with Lm2. (E) juice E with Lm14. Numerical labels represent signals used for ROIs-based analysis, corresponding to those in Table 1. (PDF) [file pone.0182229.s001.pdf]

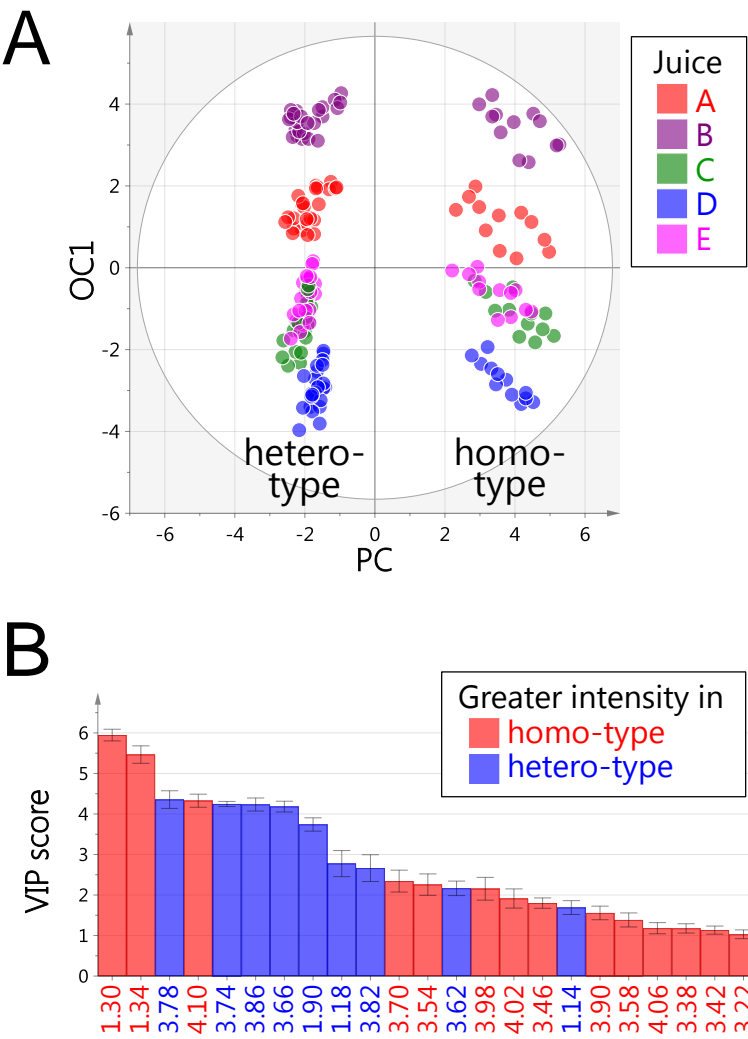

Supplement: S2 Fig — The model was evaluated by leave-one-out cross validation, providing determinant coefficient (R2) and cross-validation determination coefficient (Q2) of 0.965 and 0.951, respectively. (A) Score plot color-coded according to juices. Predictive component (PC) and first orthogonal component (OC1) represent 43.1% and 29.9% of the total variance, respectively. (B) VIP scores. Metabolites with a score >1.0 are shown in descending order. Red and blue bars show higher levels in the samples of homo- and hetero-fermentative strains. Variable labels represent central chemical shifts of each bin (0.04-ppm width). (PDF) [file pone.0182229.s002.pdf]

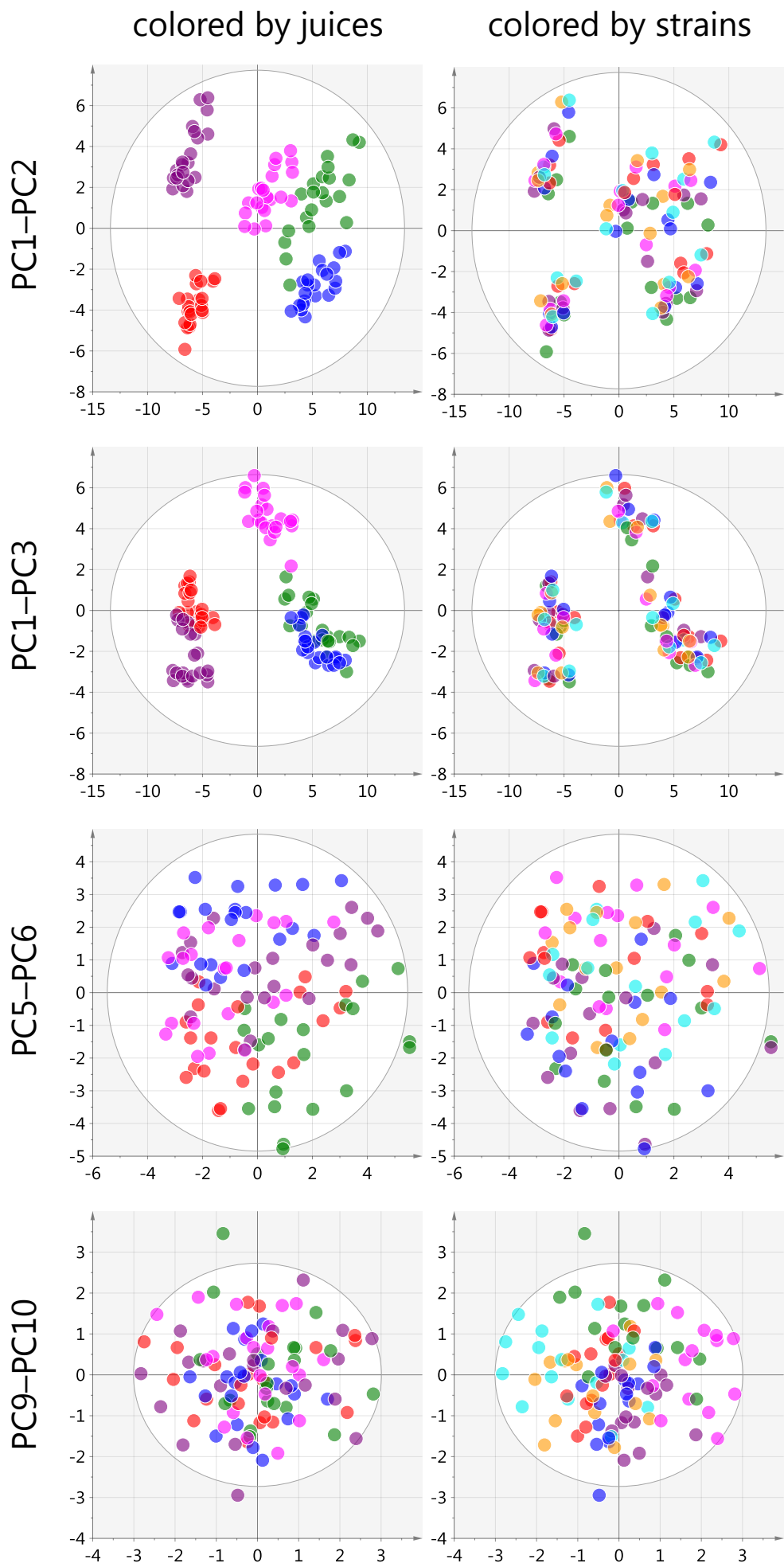

Supplement: S3 Fig — Color code corresponds to Fig 3. The ten principal components explained the total variance as follows: PC1, 38.1%; PC2, 12.7%; PC3, 9.4%; PC4, 7.1%; PC5, 6.4%; PC6, 5.0%; PC7, 4.5%; PC8, 3.6%; PC9, 1.9%; PC10, 1.6%. (PDF) [file pone.0182229.s003.pdf]
