# Supplementary material for: Rapid discrimination of strain-dependent fermentation characteristics among Lactobacillus strains by NMR-based metabolomics of fermented vegetable juice
Source: PLoS One. 2017 Jul 31;12(7):e0182229. doi: 10.1371/journal.pone.0182229 (PMC5536307; doi:10.1371/journal.pone.0182229)
Supplement: S1 Table — (PDF) [file pone.0182229.s004.pdf]

S1 Table. Nutritional composition of vegetable juice products used in this study.

juice	nutrition in 100 mL					
	energy (kcal)	protein (g)	lipid (g)	carbohydrate (g)	dietary fiber (g)	sodium (mg)
A	35	0.4	0	7.8	0.4	25
B	40	0.4	0	9.5	0.3	35
C	36	1.1	0	7.3	1.0	46
D	26	1.0	0	5.0	0.8	24
E	35	1.0	0	7.5	0.5	62
